# Association between Exposure to Environmental Tobacco Smoke and Biomarkers of Oxidative Stress Among Patients Hospitalised with Acute Myocardial Infarction

**DOI:** 10.1371/journal.pone.0081209

**Published:** 2013-12-05

**Authors:** Ian L. Megson, Sally J. Haw, David E. Newby, Jill P. Pell

**Affiliations:** 1 Free Radical Research Facility, Department of Diabetes and Cardiovascular Science, University of Highlands and Islands, Inverness, United Kingdom; 2 School of Nursing, Midwifery and Health, University of Stirling, Stirling, United Kingdom; 3 Centre for Cardiovascular Science, University of Edinburgh, United Kingdom; 4 Institute of Health and Wellbeing, University of Glasgow, Glasgow, United Kingdom; University of Rochester Medical Center, United States of America

## Abstract

**Objective:**

To determine whether exposure to environmental tobacco smoke was associated with oxidative stress among patients hospitalised for acute myocardial infarction.

**Design:**

An existing cohort study of 1,261 patients hospitalised for acute myocardial infarction.

**Setting:**

Nine acute hospitals in Scotland.

**Participants:**

Sixty never smokers who had been exposed to environmental tobacco smoke (admission serum cotinine ≥3.0 ng/mL) were compared with 60 never smokers who had not (admission serum cotinine ≤0.1 ng/mL).

**Intervention:**

None.

**Main outcome measures:**

Three biomarkers of oxidative stress (protein carbonyl, malondialdehyde (MDA) and oxidised low-density lipoprotein (ox-LDL)) were measured on admission blood samples and adjusted for potential confounders.

**Results:**

After adjusting for baseline differences in age, sex and socioeconomic status, exposure to environmental tobacco smoke was associated with serum concentrations of both protein carbonyl (beta coefficient 7.96, 95% CI 0.76, 15.17, p = 0.031) and MDA (beta coefficient 10.57, 95% CI 4.32, 16.81, p = 0.001) but not ox-LDL (beta coefficient 2.14, 95% CI −8.94, 13.21, p = 0.703).

**Conclusions:**

Exposure to environmental tobacco smoke was associated with increased oxidative stress. Further studies are requires to explore the role of oxidative stress in the association between environmental tobacco smoke and myocardial infarction.

## Introduction

Exposure to environmental tobacco smoke (ETS) is associated with an increased risk of developing cardiovascular disease. A meta-analysis, conducted in 1999, reported a pooled relative risk of 1.25 (95% CI 1.17, 1.32), and demonstrated a dose-response relationship [1]. Even brief exposure to ETS can produce changes in platelet activation and endothelium-dependent vasodilation [2] of a comparable magnitude to those observed in active smokers [3]. Side-stream smoke contains higher concentrations of toxic gases and small, respirable particles than mainstream smoke [4]. Therefore, ETS exposure carries up to 90% of the risk of cardiovascular events associated with active smoking [5].

ETS exposure is also associated with cardiovascular disease progression. In the apolipoprotein-E deficient murine model, exposure to ETS for 21 days increased atherosclerotic lesions by 76% [5], and in a general population cohort, those exposed to ETS had 20% higher progression of carotid intima media thickness over three years [5]. Animal experiments suggest that ETS exposure results in larger infarctions and an increased risk of ventricular hypertrophy [6], [7], with evidence of a dose response in the former [7]. ETS is associated with prognosis following acute coronary events. In a cohort of 2,172 patients admitted for either myocardial infarction or unstable angina, those exposed to ETS had higher concentrations of both troponin I and creatine kinase and were at higher risk of adverse events (death or rehospitalisation) within 30 days (adjusted RR 1.61, 95% CI 1.14–2.8) [8]. There was a dose-response in relation to the number of years the index cases had lived with their smoking partner [8]. We previously used data on never smokers, from an existing cohort of patients hospitalized for acute myocardial infarction, to investigate the association between ETS exposure and early adverse events [9]. ETS exposure was associated with increased risk of all-cause deaths, cardiovascular deaths, and fatal/non-fatal acute myocardial infarctions within 30 days of admission, with clear evidence of dose relationships for all three [9]. It is plausible that oxidative stress may be involved in the mechanism by which ETS exposure increases risk. Therefore, the aim of the current study was to use the same cohort to investigate whether there was an association between ETS exposure and oxidative stress.

## Methodology

### Main study

Written, informed consent was obtained from participants. Approval for the consent process and overall study were obtained from the West of Scotland Multi-Centre Research Ethics Committee. Written, informed consent was obtained to undertake the interviews and perform assays on the residual serum from clinical blood samples taken on admission to hospital. The multi-centre research ethics committee also approved inclusion of patients who died before consent could be obtained; including use of their blood samples and extraction of information from their case-notes.

We previously recruited a cohort of patients admitted with acute myocardial infarction to nine Scottish, acute hospitals between 2005 and 2007 [10]. Acute myocardial infarction was defined as a detectable cardiac troponin I or T concentration in a patient admitted as an emergency for chest pain. Over the study period, troponin assays were performed routinely on all patients admitted to Scottish hospitals with chest pain. Therefore, this case definition could be applied consistently across all hospitals and on all patients, irrespective of date, time or route of admission. We excluded patients with other causes of raised troponin (types 2–5 myocardial infarction): renal failure, thromboembolic disease, myocarditis and coronary revascularisation.

In order to ensure complete case ascertainment, the hospital laboratories produced daily lists of patients with detectable serum troponin concentrations. Dedicated research nurses identified all eligible patients on the lists and completed structured interviews to obtain information on age, sex and postcode of residence, as well as self-reported smoking status and information on ETS exposure. In Scotland, there are 6,505 datazones, based on postcode of residence, with a mean population of 800. The Scottish Index of Multiple Deprivation (SIMD) for each datazone is derived from information on income, employment, health, education (including skills and training), housing, crime, and access to services (http://www.scotland.gov.uk/Topics/Statistics/SIMD). The SIMD has been used to derive quintiles of socioeconomic status for the Scottish population; ranging from 1 (most deprived) to 5 (least deprived). We used postcode of residence to categorize our study participants according to these quintiles.

All samples were centrifuged and stored locally at −20°C before being transported on dry ice. One aliquot from each patient was sent to the ABS Laboratory (London, UK) where cotinine assays were performed using gas chromatography with a specific nitrogen/phosphorous detector GC-NPD. Cotinine and the internal standard 5-methyl cotinine were extracted using dichloroethane from 100 µL of serum after alkalisation using sodium hydroxide. The lower limit of detection was 0.1 ng/mL.

### Sub-study

In the current study, we compared the 60 patients who classified themselves as never smokers, had a serum cotinine concentration of ≤0.1 mg/mL and for whom a second aliquot of serum was available, with a randomly selected 60 patients from the 369 patients who classified themselves as never smokers, had a serum cotinine concentration of ≥3.0 mg/mL and ≤12 ng/mL and for whom a second aliquot was available. Three biomarkers of oxidative stress (protein carbonyls, malondialdehyde (MDA) and oxidised LDL (ox-LDL)) were assayed by the Free Radical Research laboratory (University of the Highlands & Islands, Inverness, UK) who were blinded to participants' cotinine concentration. MDA and ox-LDL assays were performed on all 120 samples. There was insufficient sample available to perform protein carbonyl on 14 of the 120 samples.

#### Malondialdehyde (MDA)

MDA was measured in defrosted samples using a commercially available kit (Cell Biolabs Incorporated, cat 10005020). The samples (100 µL) and standards were treated with butylated hydroxytoluene prior to incubation with SDS lysis solution (5 min, room temperature) and addition of TBA reagent (45 min, 95°C). After cooling and centrifugation (15 min, 5000 *g*), supernatant was aspirated and treated with an equivalent volume of butanol prior to further centrifugation (5 min, 10,000 *g*). The sample was then transferred to a 96-well microplate and absorbance read at λ = 532 nm; quantification was achieved against a standard curve (0–125 µM).

#### Oxidised low density lipoprotein (Ox-LDL)

Oxidised LDL was assayed using a competitive enzyme linked immunosorbant assay (Mercodia, Cat 10-1158-01). Oxidised LDL in the sample competes with a fixed amount of oxidised LDL bound to the microtiter well for the binding of biotin-labelled specific antibodies (4E6). After washing to remove unreactive sample components, the biotin labelled antibody bound to the well is detected by HRP-conjugated streptavidin, detected by reaction with 3,3′5,5′-tetramethylbenzidine. The reaction is stopped by adding acid to give a colorimetric endpoint that is read spectrophotometrically at λ = 450 nm. The results were calculated by expressing calibrators and standards as a percentage inhibition of the mean absorbance of the maximum binding at zero inhibition (B_0_). Cubic spline regression analysis was used to construct the standard curve.

#### Protein carbonyls (PCs)

PCs were assayed in defrosted samples (100 µL) using a commercially available kit (Cayman assay kit; cat 10005020). Briefly, the assay measures the reaction of PCs with 2,4-dinitrophenylhydrazine (DNPH) reaction to form a Schiff base and the corresponding hydrazine. The amount of protein–hydrozine produced is quantified spectrophotometrically (λ = 375 nm).

### Statistical analyses

Mann Whitney U tests were used to compare the concentrations of oxidative stress markers between never smokers with high and low exposure to ETS. Separate linear regression models were used to determine whether ETS exposure was associated with each of the oxidative stress biomarkers. Multivariate analyses were used to determine whether any associations remained after adjusting for the potential confounding effects of age, sex and socioeconomic deprivation. All tests were two-sided and statistical significance was defined as p<0.05.

## Results

**Figure 1:**
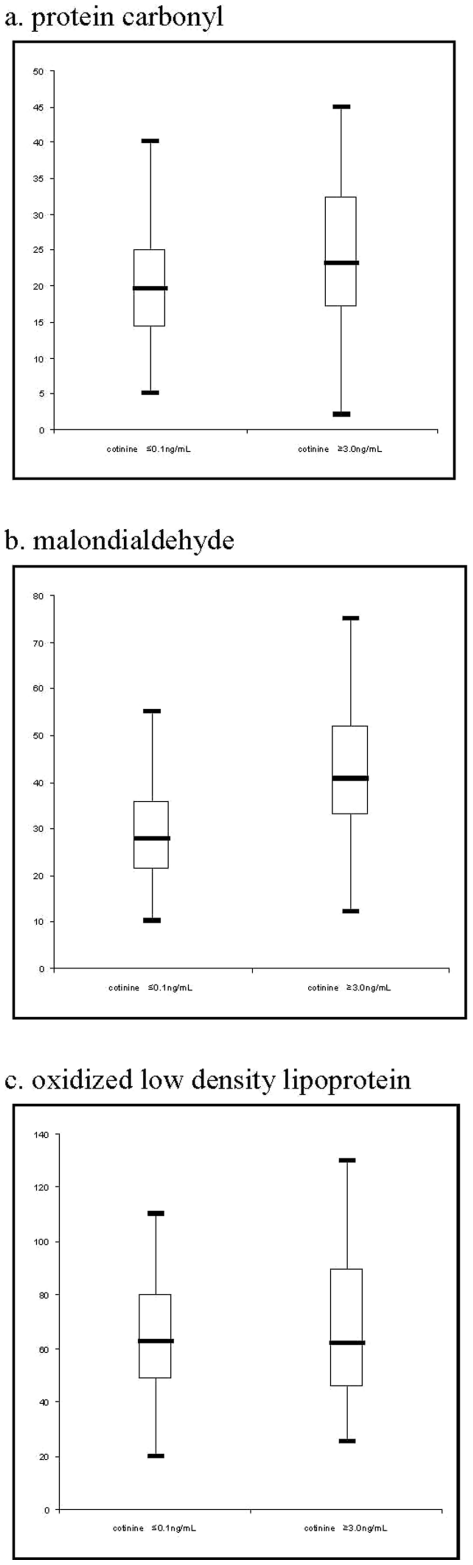
Box and whisker plots of three biomarkers of oxidative stress by exposure to environmental tobacco smoke.

Never smokers who had high levels of ETS exposure were significantly more likely to live in socioeconomically deprived areas (Table 1). They were older and more likely to be female but neither reached statistical significance (Table 1). Exposed individuals had significantly higher concentrations of plasma MDA (Table 1 & Figure 1). Their higher concentrations of plasma protein carbonyl were of borderline statistical significance and there was no significant difference in plasma ox-LDL concentrations (Table 1).

**Table 1:** Summary characteristics of patients hospitalised for myocardial infarction categorised by exposure to environmental tobacco smoke.

	Cotinine ≤0.1	Cotinine ≥3.0	P value
	N = 60	N = 60	
	N (%)	N (%)	
Sex			
Male	43 (72)	39 (65)	0.434
Female	17 (28)	21 (35)	
SIMD5			
1 (deprived)	15 (25)	42 (70)	<0.001
2	10 (17)	10 (17)	
3	8 (13)	2 (3)	
4	11 (18)	3 (5)	
5 (affluent)	16 (27)	3 (5)	
	**Median (IQR)**	**Median (IQR)**	
Age (years)	68 (57, 78)	72 (62, 75)	0.149
Protein carbonyl (units)	19.49 (14.22, 25.16)	22.99 (17.05, 32.49)	0.052
MDA (units)	27.65 (21.26, 35.86)	40.59 (32.83, 51.88)	<0.001
Ox LDL (units)	62.19 (48.15, 79.96)	61.61 (45.76, 89.44)	0.586

MWU, chi square for trend.

N number; SIMD5 Scottish Index of Multiple Deprivation quintile; MDA; LDL; IQR interquartile range.

After adjusting for the potential confounding effects of age, sex and socioeconomic status, exposure to environmental tobacco smoke was associated with serum concentrations of both protein carbonyl (beta coefficient 7.96, 95% CI 0.76, 15.17, p = 0.031) and MDA (beta coefficient 10.57, 95% CI 4.32, 16.81, p = 0.001) but not ox-LDL (beta coefficient 2.14, 95% CI −8.94, 13.21, p = 0.703) (Table 2).

**Table 2:** Univariate and multivariate linear regression analyses of the association between exposure to environmental tobacco smoke and three biomarkers of oxidative stress.

	Univariate			Multivariate*		
	Beta coeff	95% CI	P value	Beta coeff	95% CI	P value
Protein carbonyl	6.78	0.52, 13.04	0.034	7.96	0.76, 15.17	0.031
MDA	12.82	7.30, 18.34	<0.001	10.57	4.32, −16.81	0.001
Ox LDL	4.02	−5.72, 13.76	0.415	2.14	−8.94, 13.21	0.703

* adjusted for age, sex, SIMD5.

## Discussion

Among patients admitted to hospital for acute myocardial infarction, exposure to ETS was associated with increased concentrations of two plasma markers of oxidative stress (protein carbonyls and MDA). Future studies are required to determine whether oxidative stress plays a causal role in the association between ETS and myocardial infarction.

Several observational studies have demonstrated an association between exposure to ETS and oxidative stress among children. Kosecik et al. demonstrated a significantly lower total anti-oxidative response among 83 children aged 9–13 years who were exposed to ETS at home than among 61 children of similar age who were not exposed [11]. Aycicek et al. studied 84 infants under 28 weeks of age [12]. Those infants who were exposed to ETS at home had significantly lower concentrations of plasma vitamin C, thiol, TAC 1 and TAC 2 concentrations and significantly higher concentrations of plasma total peroxide, OSI1 and OSI2. Zalata et al. compared 23 exposed children under 8 years of age with 23 who were not exposed [13]. The former had significantly higher concentrations of MDA and significantly lower concentrations of glutathione peroxidise and tocopherol fractions, with evidence of dose response relationships. Aycicek and Ipek examined fetal cord blood and reported significantly higher lipid hydroperoxide, total oxidant status and oxidative stress indices among women who were exposed to ETS [14]. Shermatov et al. studied the peripheral lymphocytes of 27 children who had been exposed to ETS and 27 who had not [15]. The former had significantly higher “total oxidant status”, oxidative stress indices and DNA damage assessed by alkaline comet assay. In a study by Kim et al., there was a significant, independent association between urinary MDA and PM_2.5_ among elderly subjects but not children, suggesting that the effect of ETS exposure on oxidative stress may increase with age [16]. In 1998, Howard et al. studied non-smokers who were not exposed to ETS at home [5]. Those who were exposed to ETS in the workplace had higher concentrations of catalase and blutathione peroxidise and higher oxidative DNA mutagen 8-hydroxy-2′-deoxyguanosine.

Two intervention studies have examined the effect of ETS exposure on oxidative stress. Kato et al. exposed 30 healthy Japanese men, aged 25–39 years, to ETS for 30 minutes [17]. Exposure had no significant effect on the 15 smokers. In the 15 non-smokers, percentage flow-mediated vasodilation decreased and plasma 8-isoprostane increased significantly to concentrations comparable to those of the active smokers. Kostikas et al undertook a randomised controlled cross-over trial in which they exposed 18 never smokers to bar/restaurant levels of ETS for one hour [18]. Following exposure, H_2_O_2_ in the exhaled breath condensate increased and remained significantly higher four hours after exposure. In a study of 29,579 Chinese non-smokers, Clark et al. demonstrated an interaction with diet, whereby ETS exposure was associated with coronary heart disease mortality only in the sub-group with a diet low in fibre and anti-oxidants [19].

In a rat model, Valenti et al. demonstrated that the effect of ETS exposure on cardiovascular regulation was mediated via its influence on catalase activity [20]. Al-Arifa et al. demonstrated that exposing rats to ETS resulted in increased mRNA expression of oxidative stress genes as well as significant increases in a number of markers of oxidative stress (heme oxygenase 1, catalase, cyclooxygenase and glutathione S-transferase) [21]. Raji et al. showed that exposure of rat aortic rings to cigarette smoke impaired nitric oxide-mediated endothelial function via increased generation of superoxide anion [22]. In a subsequent in vitro study, using bovine, rat and human tissues, they demonstrated that the thiol-reactive stable compounds in cigarette smoke activated NADPH oxidase and increased endothelial superoxide anion production, thereby reducing NO bioactivity and resulting in endothelial dysfunction [23]. Based on rat models, Gentner and Weber postulated that increased lung neutrophils or pulmonary CYP1A1 may be responsible for the increase in oxidative stress following exposure to ETS and may, in turn, be related to the observed failure of blood pressure to fall during periods of sleep and a possible increase in arterial stiffness [24].

To the best of our knowledge, this is the first study of the association between exposure to ETS and oxidative stress among patients suffering acute myocardial infarction. By its very nature, oxidative stress is a difficult parameter to measure effectively in vivo. Oxidative damage to biomolecules is an accepted surrogate measure of oxygen-centred free radical generation and a range of plasma markers of lipid and protein peroxidation have been identified [25]. However, there is considerable debate as to the benefits of one measure over another and, in studies where more than one marker has been measured, there are not necessarily consistent effects across different markers of oxidative stress in response to the same stimulus, suggesting that different markers might reflect disturbances in different processes that contribute to oxidative stress. With this in mind, our approach was to measure a range of markers to provide as broad a picture as possible, whilst at the same time potentially contributing additional information as to the mechanisms involved in the process. Three markers were selected: MDA and ox-LDL as markers of lipid peroxidation, and protein carbonyls as a marker of protein oxidation. MDA is an end-product of lipid peroxidation that has been associated with a range of risk factors for cardiovascular disease, including cigarette smoke [26]. Ox-LDL is a key mediator of atherosclerosis and plasma concentrations have been found to be elevated in a range of cardiovascular diseases [26], [27]. Plasma ox-LDL and MDA concentrations may also be associated with plaque instability [27], [29]; with studies suggesting higher concentrations among patients presenting with acute MI compared to those with stable angina [27], [28] and, among patients with coronary artery disease, MDA concentration are higher in patients with thin-cap atheroma and complex plaques [29]. Protein carbonyls, on the other hand, represent oxidation in a different compartment in the plasma that have been shown to be associated with smoking in some studies [30] and with age, ethnicity and some socio-economic factors amongst smokers, but not with urinary cotinine in another [31]. Given the relevance of these three markers in our patient group, it was considered sensible to measure them all where sufficient sample was available.

The lack of association of cigarette smoke exposure with ox-LDL is perhaps surprising, given that previous studies have shown an association of this marker with both smoking and with acute MI, but this marker is perhaps the most susceptible to dietary influences, such as recent meals containing cholesterol or oxidised cholesterol, which could not be controlled for in this study. Nevertheless, the finding that other markers of oxidative stress in the lipid (MDA) and aqueous (protein carbonyls) plasma compartments both show an association with ETS exposure is supportive of the notion that oxidative stress caused by smoke inhalation is implicated in acute MI in vulnerable individuals. Furthermore, the discrepancy in the findings between different markers of oxidative stress highlights the need for multiple markers to be measured, given that each will be the product of different processes with different formation and clearance rates. In our analyses we were able to adjust for the potential confounding effects of age, sex and area deprivation. However, we did not have access to information on lifestyle risk factors such as physical activity, diet and adiposity. Therefore, residual confounding is possible. Our findings were based on relatively small numbers of patients and merit corroboration in larger studies.

### Conclusion

Oxidative stress increases the risk of developing cardiovascular disease and disease progression and it is now well established that active smoking predisposes to oxidative stress. Our study adds to increasing evidence that ETS exposure may also be associated with oxidative stress. This mechanism may role a play in the association between ETS exposure and myocardial infarction.
